# How to squat? Effects of various stance widths, foot placement angles and level of experience on knee, hip and trunk motion and loading

**DOI:** 10.1186/s13102-018-0103-7

**Published:** 2018-07-17

**Authors:** Silvio Lorenzetti, Mira Ostermann, Fabian Zeidler, Pia Zimmer, Lina Jentsch, Renate List, William R. Taylor, Florian Schellenberg

**Affiliations:** 1 Institute for Biomechanics, ETH Zurich, Leopold-Ruzicka-Weg 4, 8093 Zürich, Switzerland; 2 Swiss Federal Institute of Sport Magglingen, SFISM, Hauptstrasse 247, 2532 Magglingen, Switzerland; 3 Department of Business, Health & Social Work, Bern University of Applied Science, Schwarztorstrasse 48, 3007 Bern, Switzerland; 4 Department of Medicine, Sports & Healthcare, University of Applied Science Technikum Vienna, Höchstädtplatz 6, 1200 Wien, Austria

**Keywords:** Squat exercise, Squatting, Knee alignment, Varus / valugus

## Abstract

**Background:**

Squatting is a core exercise for many purposes. The tissue loading during squatting is crucial for positive adaptation and to avoid injury. This study aimed to evaluate the effect of narrow, hip and wide stance widths, foot position angles (0°, 21°, and 42°), strength exercise experience, and barbell load (0 and 50% body weight, experts only) during squatting.

**Methods:**

Novice (*N* = 21) and experienced (*N* = 21) squatters performed 9 different variations of squats (3 stance widths, 3 foot placement angles). A 3D motion capture system (100 Hz) and two force plates (2000 Hz) were used to record mediolateral knee displacement (ΔD*), range of motion (RoM) at the hip and knee joints, and joint moments at the hip, knee, and lower back.

**Results:**

Both stance width and foot placement angles affected the moments at the hip and knee joints in the frontal and sagittal planes. ΔD* varied with stance width, foot placement angles and between the subjects’ level of experience with the squat exercise as follows: increasing foot angle led to an increased foot angle led to an increased ΔD*, while an increased stance width resulted in a decreased ΔD*; novice squatters showed a higher ΔD*, while additional weight triggered a decreased ΔD*.

**Conclusions:**

Suitable stance width and foot placement angles should be chosen according to the targeted joint moments. In order to avoid injury, special care should be taken in extreme positions (narrow stand-42° and wide stance-0°) where large knee and hips joint moments were observed.

**Electronic supplementary material:**

The online version of this article (10.1186/s13102-018-0103-7) contains supplementary material, which is available to authorized users.

## Background

Exercises related to movements from daily activities are of major interest within physical exercise development and research. Squatting features components of everyday functional movements such as walking, ascending and descending stairs, sitting down, and standing up [1, 2]. The squat strengthens the muscles in the lower limb and improves the ability to counteract a medial or lateral displacement of the knee [3]. Common techniques to vary the squat exercise include changes in stance width, foot placement angle, hip depth, and extra load. Similar to split squats [4], these different techniques lead to different loading conditions and movements and thus to different opinions among therapists, coaches, and experts regarding the most effective squatting execution. Besides the health benefits, and a general low injury risk of strength training compared to other sports, squatting has been identified as a strength exercise with a raised risk of injury for the lower limbs and the trunk compared to other strength exercises [5]. Evidence based guidelines exist for the execution of a squat, and these include foot stance of shoulder width or wider, maintaining the feet flat on the ground, and toes pointing forward or slightly outward by no more than 10° [6–8]. In addition, the knees should track over the toes throughout the squat motion without knee displacement either medially or laterally [7]. To create and evaluate these guidelines, a number of studies have investigated the kinematics, muscle activity, and loading conditions that occur in the lower extremities during different execution forms of the squat exercise. A comparison between the restricted knee (where the knee should not pass anteriorly of the toe) and the unrestricted knee (where the knee is free to pass beyond the toe) techniques during squatting shows that the range of motion (RoM) of the knee [9, 10] and of the lumbar and thoracic spine differs significantly and furthermore when adding a greater load [11]. With increasing load, the RoM of the lumbar curvature decreases significantly, and the thoracic curvature RoM decreases with increased additional load on the barbell from 25 to 50% of participant’s body weight [11].

The effect of foot placement angles has primarily been investigated by examining the change in electromyography muscle activity [12–16] but also by kinematic and kinetic analyses. While stance width affects muscle activity in the lower extremities, varying foot placement angles during squats does not seem to play a major role on either muscle activity or knee joint contact forces [17, 18]. In contrast, different stance widths have been found to influence the motion and joint loading of hip and knee but not the trunk motion [19, 20]. Here, it needs to be mentioned, that these authors included powerlifters that probably have acquired a different squatting strategy than observed in other athletes. Therefore, different types of executions clearly influence both musculoskeletal movement and loading conditions; thus, specific variations in squat techniques (depth, speed, stance width, and bar load) can be optimally tailored to achieve an athlete’s or patient’s training goals [8, 18].

While many published studies refer to advanced squatters such as Olympic or national weightlifters [15, 19] or powerlifters [20], the present study focusses on understanding the major influences of squat technique from a perspective of both more and less experienced participants working out in a gym. No studies have investigated a stance width below 10 cm, since most attention has been paid to shoulder or hip stance width [10, 12, 17, 19, 21].

While it is well known that a greater knee valgus angle in the knee during squatting is a risk factor for lower extremity injuries, knee displacement in the frontal plane has only been examined using cohorts with excessive medial knee displacement. Here, especially gastrocnemius muscle tightness and increased adductor activity may cause excessive mediolateral knee displacements, and squatting variations such as heel lifts or improved strength in the ankle lead to lower mediolateral movements [22–25].

While anterior-posterior translation of the knee during squats or deep knee bends has been studied [9, 10, 26, 27], the mediolateral displacement (leading to varus or valgus postures) has only been examined using cohorts with excessive medial knee displacement, showing that increasing knee valgus angles result in an increasing risk factor for injury. To lower mediolateral movements, changes in the squatting variations such as heel lifts or improving strength in the ankle is recommended [7, 22–25]. However, particularly the mediolateral movement of the knee within healthy novice and experienced strength exercise participants is missing in literature. Therefore, the aim of this study was to assess knee and hip range of motion and moments, including knee frontal plane displacement, and in addition spinal curvature, and moment at L4/L5 level, of experienced and novice squatters during different execution forms of parallel back squats.

## Methods

### Participants

Forty-two participants were recruited by email and public announcement at ETH Zurich and in surrounding fitness centres. Novice and experienced participants with good health, without a lower-limb surgery, and who regularly exercised in a fitness centre or gym were included. Participants who performed squatting exercises once a week or more, for at least 1 year, and with a one repetition maximum of at least 80% of their body weight were considered experienced squatters. All participants provided written informed consent to participate in this study, which was approved by the local ethics committee (EK 2015-N-27). The novice group included 11 women and 10 men (age 25 ± 6 years; weight 66.3 ± 11.2 kg; height 172.2 ± 8.8 cm) and the experienced group included 10 women and 11 men (age 25 ± 5 years; weight 68.9 ± 11.2 kg; height 174.0 ± 9.1 cm).

### Squat position

Three stance widths were examined: narrow stance (NS) described a stance width of 10% of the distance from the greater trochanter to the floor; hip stance (HS) was a distance between the two anterior superior iliac spines; and a wide stance (WS) was twice the distance between the anterior superior iliac spines. The HS and WS equalled the two stance widths analysed by McKean and co-workers [21]. Based on previous study results [17], three different foot angle placements were examined (0°, 21°, and 42°). The angle of each foot was defined as that between the line pointing straight ahead and the foot axis (line through the middle of the heel and the second toe). Performing each of the three stance widths with the three foot placement angles resulted in total nine different squatting positions, each completed by all the participants.

### Squat executions

After a warm up phase of 5 min, both groups performed a set of five squats in each of the nine different positions (Table 1), in a randomized order. The experienced group additionally performed squats under loaded conditions, using additional weight of 50% of their body weight on the barbell (e+). To ensure an equal arm position during the non-loaded squat performances (n and e), a very light wooden bar (less than 0.5 kg) was handed to the participants and placed on the trapezius muscle to simulate the presence of the barbell. Between each set, the participants received a two-minute rest in order to minimize possible effects of fatigue [28, 29]. For all conditions, standardized instructions were provided (Table 2).

**Table 1:**
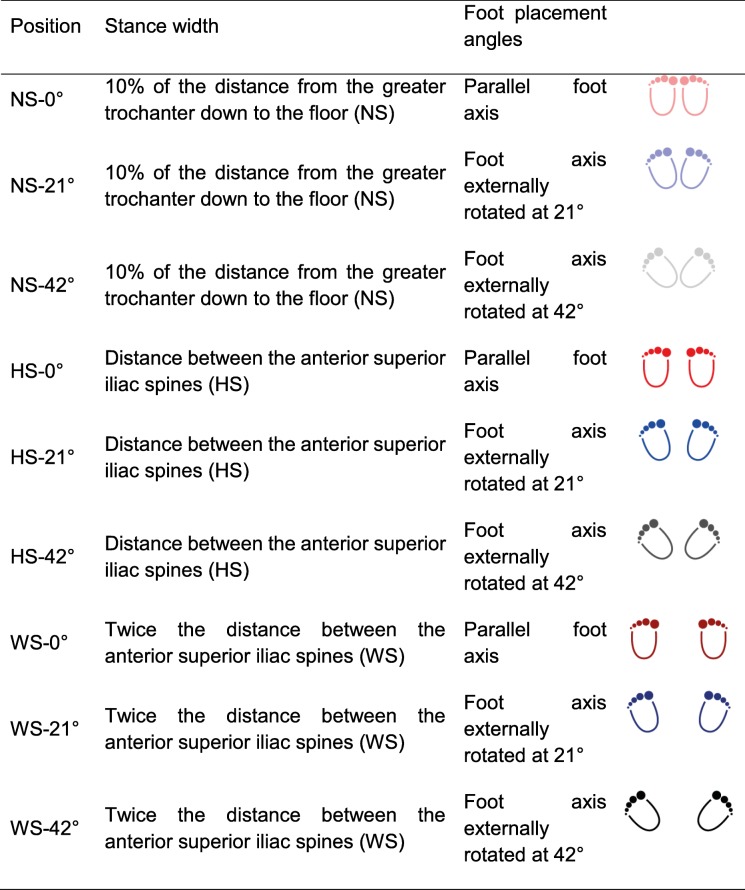
Stance width and foot placement angles for the three positions hip stance (HS), narrow stance (NS) and wide stance (WS)

**Table 2:** Standardized instructions for squat performance

Instructions	
1	Place the bar (barbell) on the trapezius muscle and hold it with a comfortable hand position.
2	Stand upright and place each foot on one of the given lines. Keep the heel and second toe aligned.
3	Keep your back straight throughout the movements.
4	Perform the squat at the same speed in the downward and upward movements.
5	Try to go as far downward as possible, at least bringing your thigh parallel to the floor.

### Procedure for data collection

The data collection took place in the Laboratory for Movement Biomechanics of the Institute for Biomechanics (IfB) at ETH Zurich between January and April 2016. For the measurement of the kinetic data, two Kistler force plates using a sampling frequency of 2000 Hz (Kistler Instrumente AG, Winterthur, Switzerland) were used, one for each foot [10]. To ensure the correct position of the feet, a laminate paper marked with the foot placement angles was attached to each force plate. Thus, the nine positions to be carried out by the participants were marked on the floor.

**Fig. 1:**
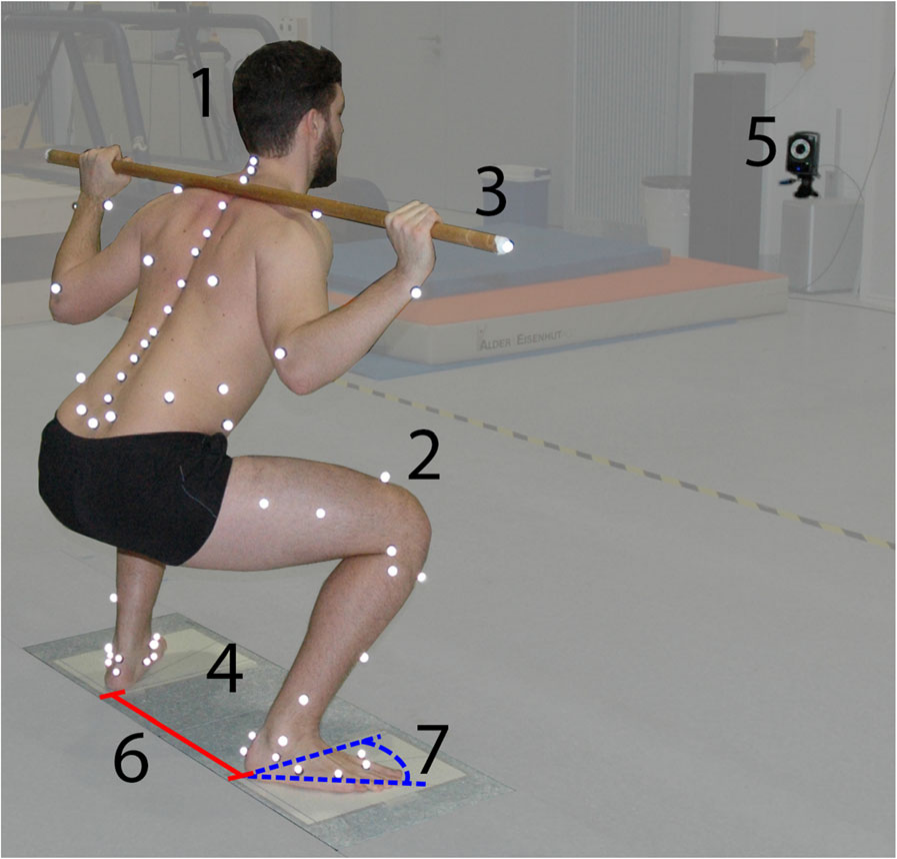
Measurement set up including the participant (1) fitted with the IfB Marker Set (2), the wooden bar (3), force plates under each foot (4) and Vicon cameras (5) for the condition wide stance (6) with a 42° (7) foot angle placement (WS-42°)

The kinematic data were gathered synchronized to the force data using the 3-dimensional motion capture system Vicon (Vicon Motion System, Oxford Metrics Ltd., UK), with 22 fixed and 7 mobile cameras (MX40 and MX160) and a sampling frequency of 100 Hz. The IfB Marker Set [11], consisting of 55 markers on the legs, pelvis, shoulder and arms, 22 on the back and 2 attached to the wooden bar or the barbell, was used (Fig. 1). Through standardized basic motion tasks, the centre and axis of the ankle, knee, and hip joints were functionally determined [11], while the joint centre of L4/L5 was defined anatomically based on anthropometric data [30]. The conventions of the joint coordinate system developed by Grood and Suntay [31] were used to describe the kinematics of these joints. For the back, both a segmental and a curvature approach was used [32, 33].

The external joint moments were calculated using an inverse approach with a quasi-static solution [34], taking the ground reaction force and kinematic data into account [35], normalized to BW and averaged over both limbs at the hip and knee joints [10, 36]. The inverse approach included the position of the joints, the forces acting on each foot, and the gravitational force of the segments [10, 36]. Due to slow accelerations of the segments during these exercises, the inertia forces were neglected. Positive values represent external flexion, adduction and internal rotation moments. All calculations were completed using MATLAB (version R2014a, The MathWorks Inc., Natick, MA, USA).

### Data analysis

A single squat cycle was defined with participants starting in an upright position, moving downwards to the lowest point possible, and returning to the upright position. The vertical velocities (v_barb_ >0.04 m/s) of the wooden bar and the barbell were tracked using the mean vertical velocity of the two markers attached to each end [11, 36]. From each squat position, the averages of five repetitions were calculated separately for each participant. Further calculations and the statistical analysis were performed using the average data from each participant.

Leg alignment was defined as the deviation of the knee joint centre (KJC) from the sagittal plane (ΔD* in % of participant’s leg length) of each leg, which was formed by the ankle joint centre (AJC), the hip joint centre (HJC), and the marker on the head of the second metatarsal (TO):

1 $$ \varDelta {D}_{right/ left}^{\ast }=\frac{\left(\left(\overrightarrow{\mathrm{AJC}-\mathrm{KJC}}\right)\frac{\overrightarrow{N}}{\mid \overrightarrow{N}\mid}\right)}{LL}\cdot 100, $$

where $\overrightarrow{N}$ is the normal vector of the sagittal plane of each leg pointing towards lateral:

2 $$ \overrightarrow{N_{left}}=\left(\overrightarrow{{\mathrm{HJC}}_{left}-{\mathrm{AJC}}_{left}}\right)\mathbf{x}\left(\overrightarrow{{\mathrm{TO}}_{left}-{\mathrm{AJC}}_{left}}\right), $$

3 $$ \overrightarrow{N_{right}}=\left(\overrightarrow{{\mathrm{TO}}_{right}-{\mathrm{AJC}}_{right}}\right)\mathbf{x}\left(\overrightarrow{{\mathrm{HJC}}_{right}-{\mathrm{AJC}}_{right}}\right), $$

And *LL* is the participant’s respective leg length, calculated as follows:

4 $$ LL=\left(\overrightarrow{\mathrm{KJC}-\mathrm{AJC}}\right)+\left(\overrightarrow{\mathrm{KJC}-\mathrm{HJC}}\right). $$

Each limb was analysed separately (ΔD*_right/left_) and normalized to each participant’s leg length. By definition, knee valgus is represented by ΔD* < 0, a straight alignment by ΔD* = 0, and knee varus by ΔD* > 0, which takes the different stand widths and foot placement angles into account [3]. Contrary to the anatomical convention and definition of knee varus and knee valgus as convex or concave movement from the medial plane, this calculation signifies that the coordinate system by Grood and Suntay [31] was also adjusted for, therefore accounting for each standing position. The lumbar curvature was calculated by fitting a circle around the skin markers in the lumbar part of the spine [37] a method that allows the quantification of the spinal dynamics during movements [11, 38–40]. An inverse dynamic approach was used to calculate the moments in the joints [11, 36, 41].

A two factor linear mixed method model was used to explore the two groups, foot placement angles, and stance width as fixed effects and participants as random effects were used to test the influence of the different execution types on the average mean knee deviation (ΔD*) between novice and experienced squatters, as well as with and without extra load within the experienced squatters. A Bonferroni post-hoc test was conducted to adjust the significance level for multiple comparisons. Descriptive analyses were conducted for all other parameters, including the average RoMs of the KJC and HJC, the RoMs of the lumbar curvature, as well as the sagittal and frontal moments of the HJC, KJC and lumbar spine. Statistical tests were performed using IBM SPSS (version 22, SPSS AG, Zürich, Switzerland).

## Results

The averaged stance widths of the investigated squat performances were for NS, 0.091 ± 0.007 m; for HS, 0.24 ±0.02 m; and for WS, 0.48 ± 0.03 m. There was no significant (*p* = 0.614) interaction between group and foot position.

### Kinematics

#### Average mean knee deviation (ΔD*)

**Fig. 2:**
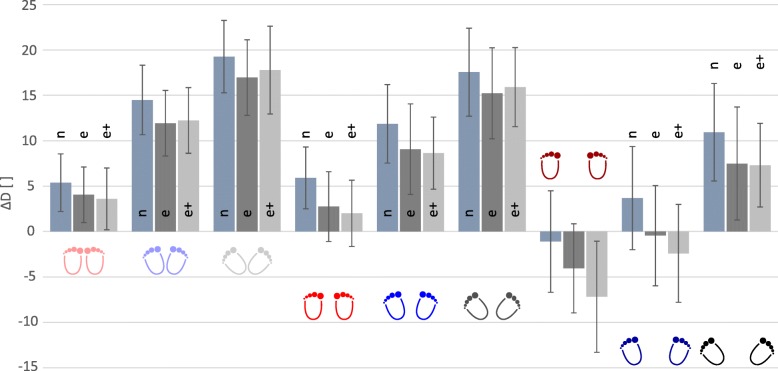
Averaged values including standard deviation of ΔD* [% of leg length] displayed for the novice squatter (n), the experienced squatter non-loaded (e) and loaded (e+), for all three stance widths and all three foot placement angles. ΔD* is significant different between the different stance widths, foot placement angles and between the groups. While an increasing angle in the foot placement angle led to an increasing ΔD*, an increased stance width resulted in a decreased ΔD*. Novice squatters showed a higher ΔD*, while additional weight provoked a smaller ΔD*

**Fig. 3:**
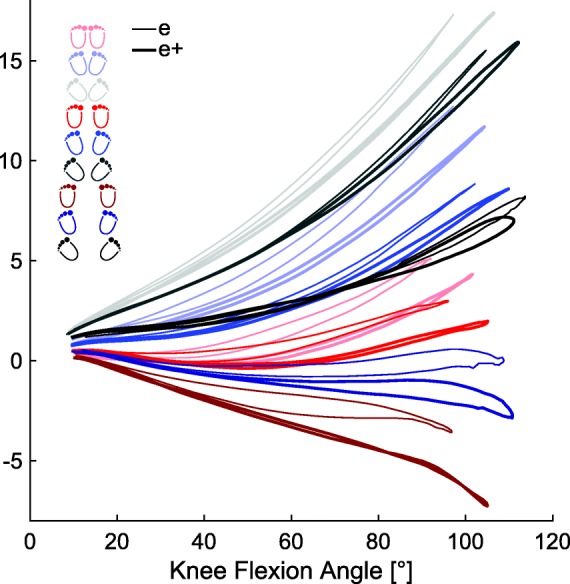
Averaged values of ΔD* [% of leg length] as a function of the knee flexion angle [°] of the experienced cohort with the wooden bar (e: thin line) and with extra load on the barbell (e+: thick line) for all nine positions

Values of ΔD* were between − 17 and 27% of participants’ leg lengths (Fig. 2) indicating valgus and varus positions. Only position WS-0° displayed a knee valgus for all three groups, while in the experienced group, the position WS-21° additionally showed a knee valgus. For WS-0°, ΔD* was − 1, − 4% and − 7% of the participant’s leg length for novice, experienced and experienced with additional load respectively, and for WS-21° -0.5% and − 2.4% of participant’s leg length for experienced without and with additional load (Fig. 2). Significant differences in ΔD* were found between the novice and the experienced squatters, between the non-load-carrying and load-carrying execution of the experienced squatters, as well as among the factors stance width and the foot placement angle. While a wider stance led to smaller ΔD*, a wider foot placement angle caused a larger ΔD*. The novice group showed a significantly higher ΔD* than the experienced squatters, while within the experienced squatters, performing squats with extra weight loading led to a smaller ΔD*, but was dependent upon the execution form. Within each single cycle, ΔD* diverged between the different positions as a function of the knee flexion angle (Fig. 3). Within a cycle, smaller stance widths and larger the foot angles resulted in a greater ΔD*, which also increased with knee flexion angle.

#### Range of motion

While the RoM of knee adduction seemed to be constant over the different foot placement angles and step widths (factor 0.9 from NS to WS and 1.4 from 0° to 42°), both, the foot placement angles and the step widths influenced the hip adduction RoM (by a factor of 1.6 from NS to WS and 3.2 from 0° to 42°) (Table 3). Similarities could be observed in the transversal RoMs of the knee and hip, where the hip RoM seemed to be more sensitive to the different foot positions. In addition, wider stance widths and larger foot angles led to higher hip RoMs in the transversal plane.

**Table 3:**
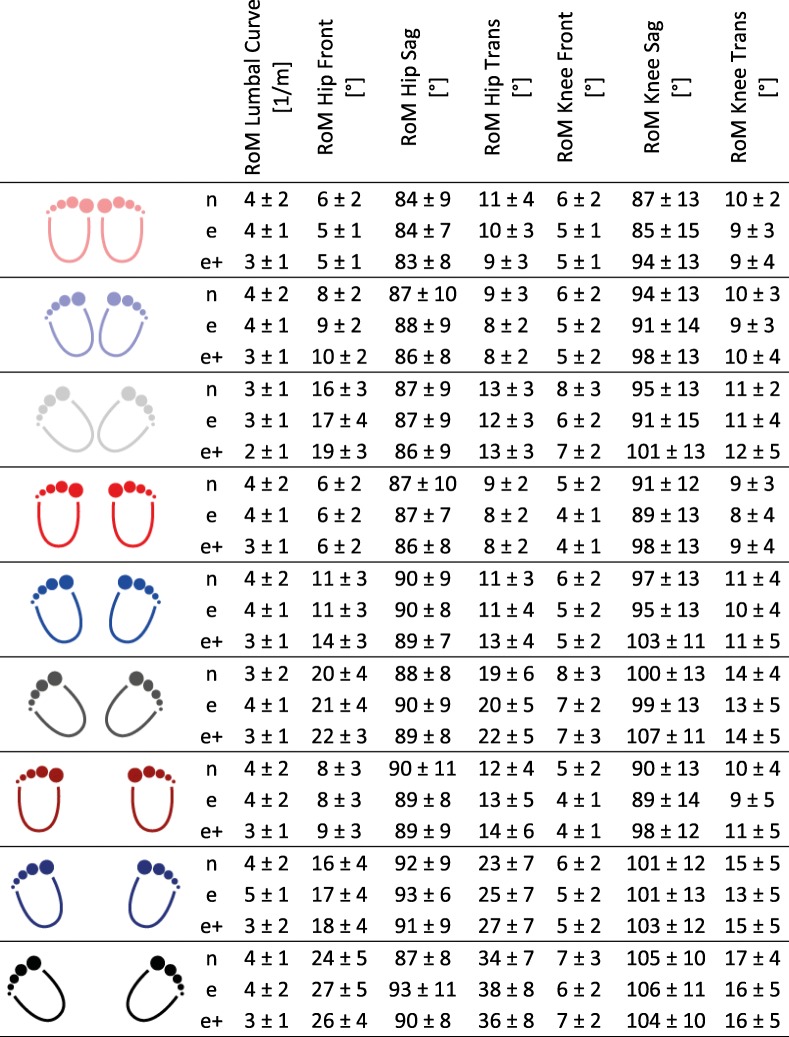
Kinematic mean values for all examined ranges of motions (RoMs), showed separately for the novice (n), the experienced (e) and the experience group with extra load (e+) for all three stance widths and all three foot placement angles

Regarding the sagittal plane, the outcomes were comparable to the other planes: A wider step width and a larger foot angle seemed to lead to higher RoM in the sagittal plane in both the hip (Additional file 1: Figure S1) and the knee (Additional file 2: Figure S2). Contrary to that, RoM in the lumbar spine appeared to be constant over the different positions, while the largest difference could be observed between the unloaded and the loaded conditions of the experienced squatter. Here, the additional load led to smaller RoMs (3.0 m^− 1^) compared to the unloaded condition (4.1 m^− 1^).

### Kinetics

All moments increased with additional load on the barbell in the experienced squatters group with a factor between 1.38 to 1.86, except the minimal external moment of the knee in the frontal plane (Additional file 3: Figure S3), which remained relatively constant and the lumbar spine moment, which increased with a factor of 1.1 only. Here, the lumbar spine moment between the different step widths and foot placement angles in the e + group varied between 1.2 and 2 Nm/kg and led to a standard deviation of 1.3 Nm/kg, which is more than 2.5 as high as the non-weighted groups (Table 4).

**Table 4:**
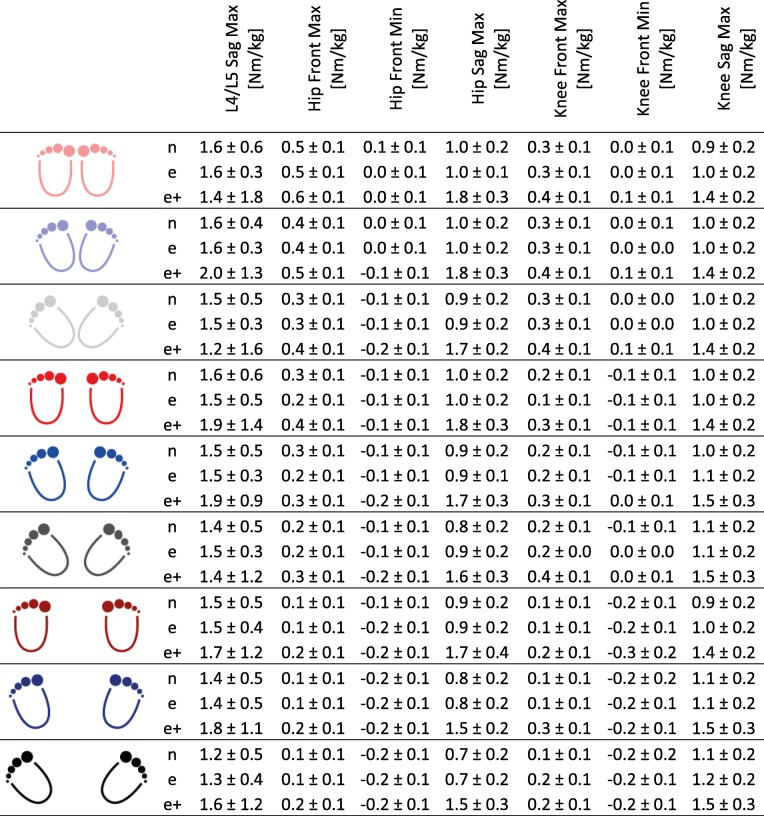
Mean values for external moments [Nm/kg], shown separately for the novice (n), the experienced (e) and the experienced group with extra load (e+), for all three stance widths and all three foot placement angles

**Fig. 4:**
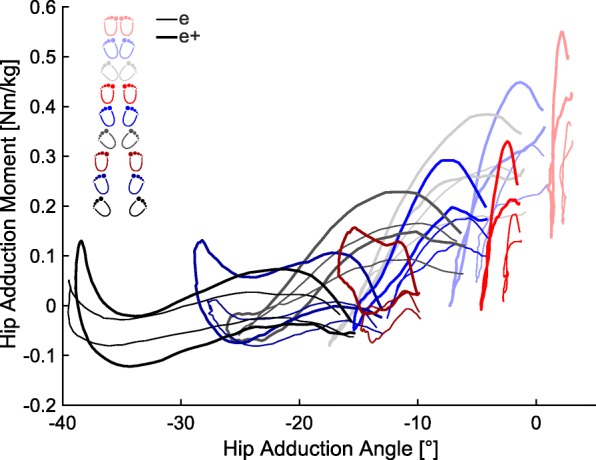
Averaged values of the external hip adduction moment [Nm/Kg] (negative: external abduction moment) as a function of the hip adduction angle [°] of the experienced cohort with the wooden bar (e: thin line) and with extra load on the barbell (e+: thick line) for all nine positions

Coupling the kinematic and kinetic values, an increased stance width and an increased foot angle led to lower maximal adduction moments in the hip with an increased hip adduction RoM (Fig. 4).

## Discussion

In this study, two cohorts (novice and experienced) were compared performing different types of squats, in order to assess the influence of stance width and foot placement on the knee and hip movement and loading. The study aimed to find a difference between novice and experienced squatters regarding the knee displacement from the sagittal plane ΔD*, as well as kinematic parameters and external joint moments in hip, knee and lower back joints in the sagittal, frontal and transversal planes. In order to assess the movement of the lumbar spine the curvature [11] was analysed. The extra load of 0% for novice and 50% for experienced squatters chosen here represents the lower end of weight used in a strength training [10] but it allows to analyse the unloaded squat movement and the effect of a moderate extra load.

### Kinematics

#### Average mean knee deviation (ΔD*)

General guidelines regarding the knee position during squat exercises recommend that the knees should be maintained vertically between the malleoli in the frontal plane, avoiding either medial or lateral knee displacement in order to reduce the risk of injury [6, 7]. Excessive mediolateral movement of the knees is thought to signal a functional deficit. These deficits can include an enhanced hip adductor activity, malfunction / weakness of the musculature of the posterior chain complex or a reduced RoM of the ankle joint, which tends to valgus positions in the knee or [7, 22–25]. However, the mediolateral displacement of the knee is not only dependent on the movement of the patella with respect to the toes in the global frontal plane, but should be analysed in the local frontal plane also, i.e. with respect to the femoral and tibial longitudinal rotation and the stance width of a squat. The parameter ΔD*, used in this study to exactly address this topic, should remain as low as possible throughout the movement to avoid displacement and additional passive forces, and thereby reduce the risk of injury.

In general, knee varus (negative ΔD*) is a much more common deficit than valgus, and a more negative ΔD* value in the novice squatters compared to the experienced ones was therefore expected. Our results demonstrate that the novice squatters tend towards a varus position, since a higher value of ΔD* was observed compared to the experienced squatters. Compared to the novice participants, the experienced cohort performed squats in all positions with a ΔD* closer to 0 except in the position WS-0°. Without any special prior instructions, all participants avoided a knee valgus or varus position in most of the squat positions, except for WS-0° and WS-21°, second for the experienced group only. However, these findings are in line with other studies, which reported a greater stretching of the lateral collateral ligament (LCL) than the medial collateral ligament (MCL), especially in an experienced group [42, 43] – thus indicating a tendency towards varus limb alignment during the task.

Special attention to the knee position should be taken when performing squats in extreme positions, since position NS-42° and WS-0° led to the highest and lowest ΔD*, respectively (Figs. 2 and 3). As a result, regarding the mediolateral displacement of the knee, we would recommend that positions HS-0° and WS-21° are employed when performing squat exercises. This is in line with literature, where it is recommended to avoid exaggerated foot placement angles in closed chain movements such as the squat [18].

Although “avoiding significant forward knee translation” and “no varus or valgus motion” is recommended by [18], the squat does not seem to compromise knee stability, and can enhance stability if performed correctly [42]. Here, our results indicate that even novice squatters are able to perform squats with a low risk of injury due to knee displacement, if extreme positions are avoided.

#### Range of motion

While the minimal curvature was measured at the widest stance and at 42° foot placement angle, spinal curvature did not seem to play a large role in joint RoMs, either in the experience of the squatters, the foot placement angle, or the stance width. On the contrary, squats should be performed with some extra load in order to maintain lumbar lordosis throughout the whole squat cycle, since the additional weight seems to enhance stabilization of the lower back. While these results are in line with previous findings [11], it is important to note that extra weight normally leads to higher moments and can cause failure due to fatigue and thus cause undesired stress on other musculoskeletal structures.

In general, other studies have recommended that an increased foot angle is used in combination with an increased stance width [17, 44]. The results of our study are somewhat contrary to these previous findings and rather indicate that a larger foot placement angle can lead to larger rotational RoMs in hip and knee, larger ab−/adduction RoMs in hip and larger flexion RoMs in knee. As a result, we would recommend that a moderate foot placement angle (approximately 20°) in combination with a moderate stance width (with feet approximately shoulder width apart) should be used.

### Kinetics

Many studies have examined the loading conditions in the lower extremities during squats. Comparisons to our study seem rather difficult, since different extra load, only 2D assessment, only one ground reaction force plate, or different calculation approaches were used [15, 45–47]. It can be assumed that a narrow stance width with a small foot angle causes a higher hip moment, while a wide stance width with a larger foot placement angle causes a higher knee moment in the sagittal plane. In the frontal plane, a narrow stance width with a small foot angle results in higher hip and knee moments. Interestingly, the knee joint moments in the frontal plane change from external abduction to external adduction within one cycle. This phenomenon is even higher with larger stance widths. By changing the joint moments, it is possible to allow a certain level of load in order to allow positive adaptation of the tissue due to the mechanical stimulus or to prevent from overload.

To our knowledge, no other study has examined the lower back moments with respect to different stance widths and foot angles during squats. Here, it is worth noting that extra weight on the barbell seems not to affect the moments in the lower back, maybe caused by the more stabilized posture, also seen in this study.

### Limitations

Several limitations existed in this study and should be mentioned. Aside the technical limitation due to the accuracy of the used measurement set up and the assumptions for the inverse dynamics, three points needs to be addressed. Firstly, the examined cohort included only healthy participants without any lower limb injuries. Thus, a transfer of the results to patients in a rehabilitation process is uncertain and should be treated with caution. Second, novice squatters examined in this study squatted under unloaded conditions only. For the experienced squatters, the 50% BW extra load was rather low, but allowed the participants to perform the squats at a low fatigue level in order to allow the acquisition with a low intra-participant variation. A comparison between different loaded conditions is required, since often additional weights are used, even throughout rehabilitation. Third, the influence of knee moments in the frontal plane on considering corresponding knee displacements is still not fully understood.

### General summary

To our knowledge, this is the first study examining the combined effects of kinematics and kinetics of the lower limb and lumbar spine during squats, and examining mediolateral knee displacement in healthy participants, while also considering the experience of the squatters. To investigate mediolateral knee movement, a new and practical approach to calculate knee displacement (ΔD*: varus and valgus postures) is presented and the results indicate that changes in foot placement angle or step width influences knee movements in the frontal plane.

## Conclusion

The knee displacement ΔD* differs significantly between the different stance widths, foot placement angles and between the groups. Novice squatters without additional weight tend towards a more varus alignment, while experienced squatters showed a lower mediolateral movement. To minimize the lumbar curvature RoM, maximize sagittal RoMs in the hip and knee with a high sagittal knee moments, WS-42° with extra weight is preferable, but leads to large RoMs in the transverse and frontal planes in the hip and knee, as well as a lower hip sagittal moment. Here, it is noteworthy that extra weight on the barbell seems not to affect the moment in the lower back.

Since limb alignment, as well as RoM of the lower extremities and the lumbar curvature are dependent on foot placement angles, the exact squat protocol should be chosen wisely, where caution should be taken when performing squats in extreme positions (NS-42° and WS-0°). Additionally, a narrow stance width with small foot angle results in increased hip and knee moments in the frontal plane.

## Additional files

Additional file 1: **Figure S1.** Averaged values of the hip moment in the sagittal plane [Nm/Kg] as a function of the hip flexion angle [°] in the experienced cohort with the wooden bar (e) and with extra load on the barbell (e+) for all nine positions. (PDF 181 kb)

Additional file 2: **Figure S2.** Averaged values of the knee moment in the sagittal plane [Nm/Kg] as a function of the knee flexion angle [°] in the experienced cohort with the wooden bar (e) and with extra load on the barbell (e+) for all nine positions. (PDF 176 kb)

Additional file 3: **Figure S3.** Averaged values of the knee moment in the frontal plane [Nm/Kg] as a function of the knee adduction angle [°] in the experienced cohort with the wooden bar (e) and with extra load on the barbell (e+) for all nine positions. (PDF 189 kb)

## Abbreviations

*ΔD**: Frontal knee displacement
AJC: Ankle joint centre
BW: Bodyweight
e +: Expert group with additional extra weight
e: Expert group
HJC: Hip joint centre
HS: Hip stance
KJC: Knee joint center
L4/L5: Level between the vertebra 4 and 5 in the lumbar spine
LCL: Lateral collateral ligament
*LL*: Leg length
MCL: Than the medial collateral ligament
n: Novice group
N: Number of participants
NS: Narrow stance
RoM: Range of motion
v_barb_: Vertical velocity of the barbell
WS: Wide stance
