# Correction to: How to squat? Effects of various stance widths, foot placement angles and level of experience on knee, hip and trunk motion and loading

**DOI:** 10.1186/s13102-020-0160-6

**Published:** 2020-01-29

**Authors:** Silvio Lorenzetti, Mira Ostermann, Fabian Zeidler, Pia Zimmer, Lina Jentsch, Renate List, William R. Taylor, Florian Schellenberg

**Affiliations:** 1 Institute for Biomechanics, ETH Zurich, Leopold-Ruzicka-Weg 4, 8093 Zürich, Switzerland; 2 Swiss Federal Institute of Sport Magglingen, SFISM, Hauptstrasse 247, 2532 Magglingen, Switzerland; 3 Department of Business, Health & Social Work, Bern University of Applied Science, Schwarztorstrasse 48, 3007 Bern, Switzerland; 4 Department of Medicine, Sports & Healthcare, University of Applied Science Technikum Vienna, Höchstädtplatz 6, 1200 Wien, Austria

**Correction to: BMC Sports Sci Med Rehabil (2018) 10:14**

**https://doi.org/10.1186/s13102-018-0103-7**

Following publication of the original article [1], the authors reported an error in the following sentence on page 8: “In general, knee varus (negative ΔD*) is a much more common deficit than valgus, and a more negative ΔD* value in the novice squatters compared to the experienced ones was therefore expected.”

The correct sentence reads as follows: “In general, knee varus (positive ΔD*) is a much more common deficit than valgus, and a more positive ΔD* value in the novice squatters compared to the experienced ones was therefore expected.”

Thanks to Moshe Marko for noticing this error.
