# Supplementary figures and images for: How to squat? Effects of various stance widths, foot placement angles and level of experience on knee, hip and trunk motion and loading
Source: BMC Sports Sci Med Rehabil. 2018 Jul 17;10:14. doi: 10.1186/s13102-018-0103-7 (PMC6050697; doi:10.1186/s13102-018-0103-7)

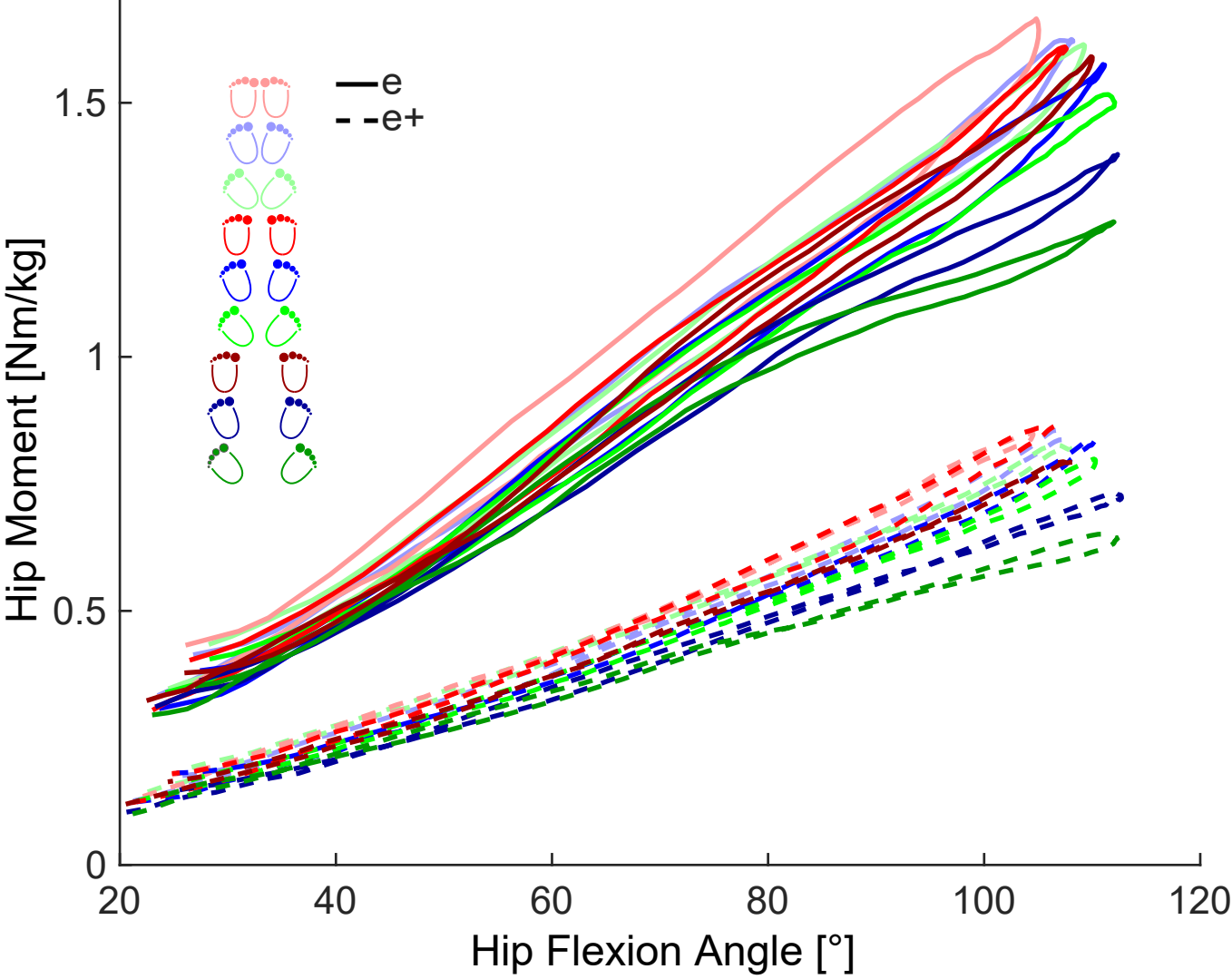

Supplement: Supplementary file 1 — Figure S1. Averaged values of the hip moment in the sagittal plane [Nm/Kg] as a function of the hip flexion angle [°] in the experienced cohort with the wooden bar (e) and with extra load on the barbell (e+) for all nine positions. (PDF 181 kb) [file 13102_2018_103_MOESM1_ESM.pdf]

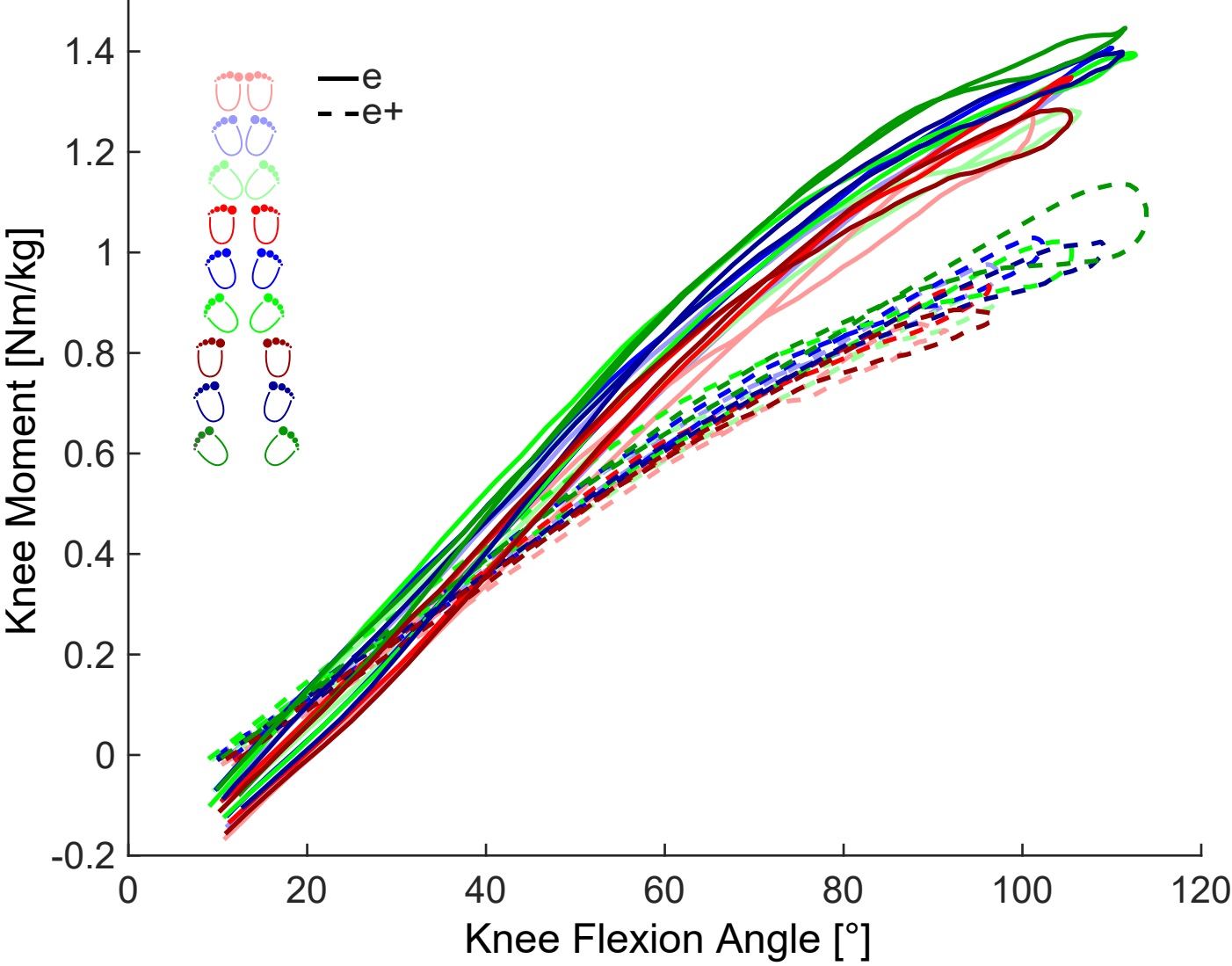

Supplement: Supplementary file 2 — Figure S2. Averaged values of the knee moment in the sagittal plane [Nm/Kg] as a function of the knee flexion angle [°] in the experienced cohort with the wooden bar (e) and with extra load on the barbell (e+) for all nine positions. (PDF 176 kb) [file 13102_2018_103_MOESM2_ESM.pdf]

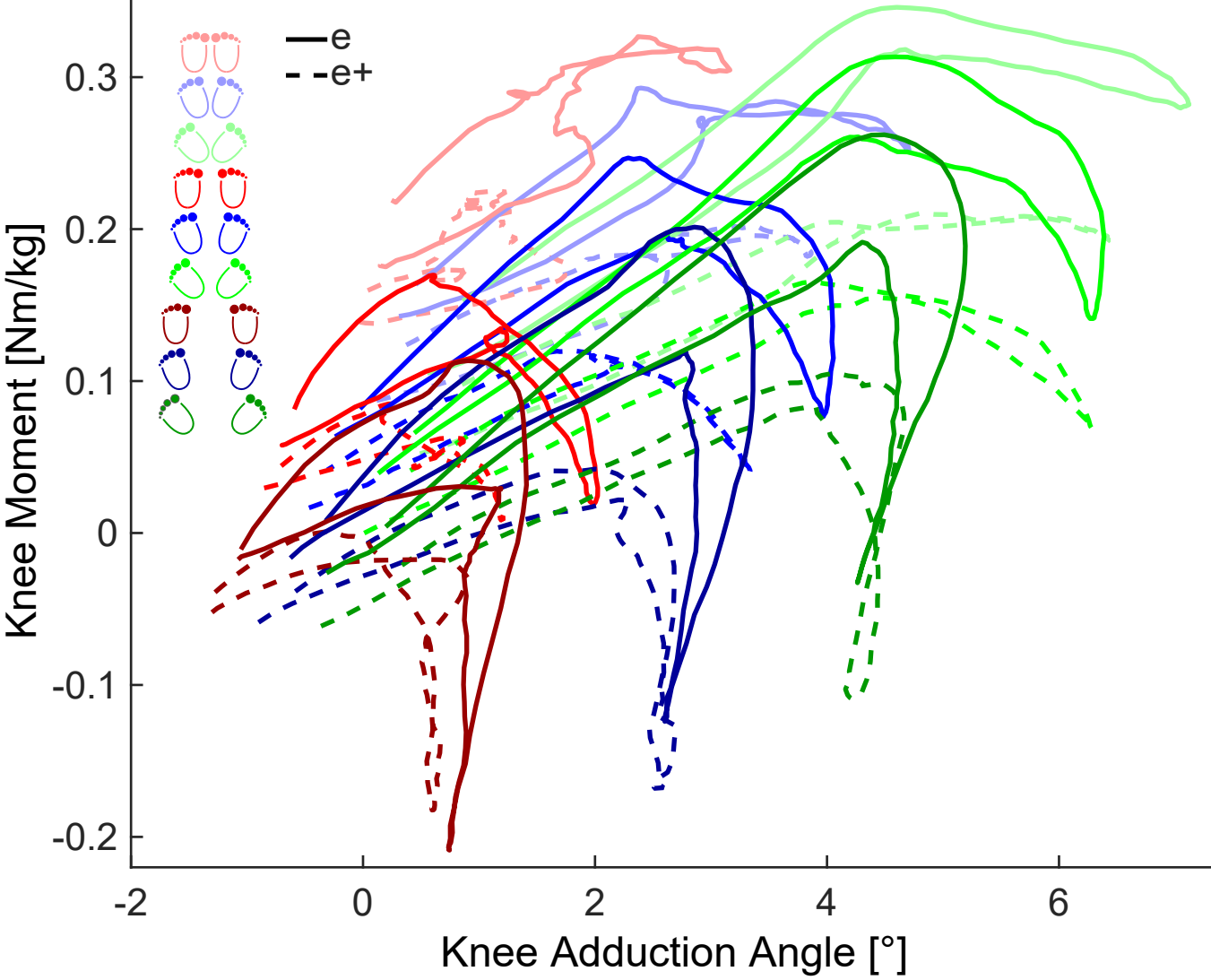

Supplement: Supplementary file 3 — Figure S3. Averaged values of the knee moment in the frontal plane [Nm/Kg] as a function of the knee adduction angle [°] in the experienced cohort with the wooden bar (e) and with extra load on the barbell (e+) for all nine positions. (PDF 189 kb) [file 13102_2018_103_MOESM3_ESM.pdf]
